# Supplementary material for: Targeted Self-Emulsifying Drug Delivery Systems to Restore Docetaxel Sensitivity in Resistant Tumors
Source: Pharmaceutics. 2022 Jan 26;14(2):292. doi: 10.3390/pharmaceutics14020292 (PMC8876228; doi:10.3390/pharmaceutics14020292)
Supplement: Supplementary file 1 [file pharmaceutics-14-00292-s001.zip › pharmaceutics-1544139.pdf]

Supplementary Material

Targeted self-emulsifying drug delivery systems to restore docetaxel sensitivity in resistant tumors

Virginia Campani[1§], Iris Chiara Salaroglio[2§], Valeria Nele[1], Joanna Kopecka[2], Andreas Bernkop-Schnürch[3], Chiara Riganti[2] and Giuseppe De Rosa[1*]

[1] Department of Pharmacy, University of Naples Federico II, Via Domenico Montesano 49, 80131 Naples, Italy. E-mail: virginia.campani@unina.it; valeria.nele@unina.it; gderosa@unina.it
[2] Department of Oncology, University of Torino, Via Santena 5/bis, 10126 Torino, Italy. E-mail: irischiara.salaroglio@unito.it; joanna.kopecka@unito.it; chiara.riganti@unito.it
[3] Department of Pharmaceutical Technology, Institute of Pharmacy, University of Innsbruck, Innrain 80/82, Innsbruck, Austria. E-mail: andreas.bernkop@uibk.ac.at
§ These authors contributed equally
* Correspondence: gderosa@unina.it

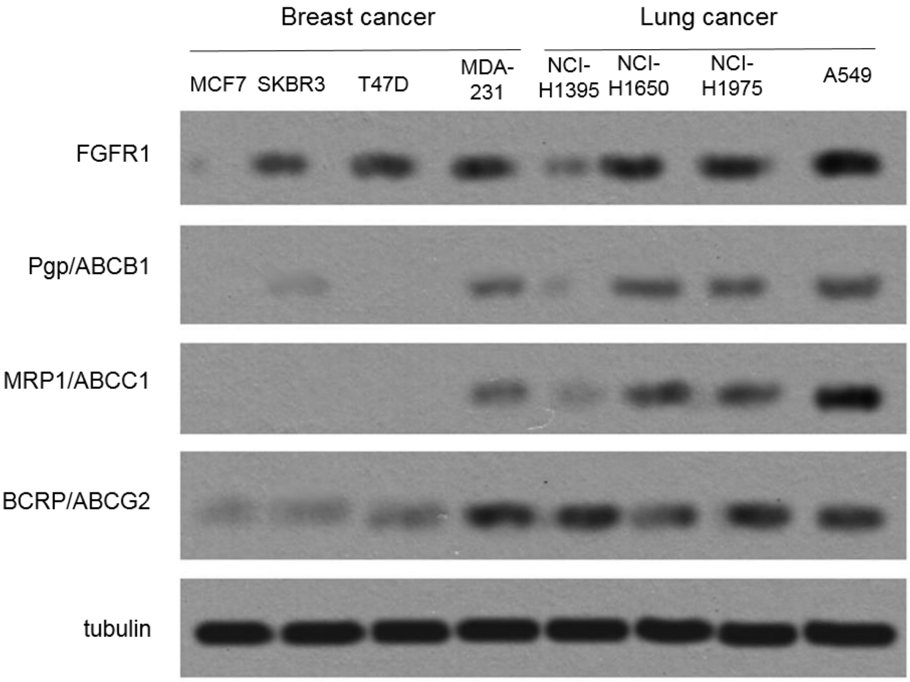

Figure S1. Expression of FGFR1 and ABC transporters in breast and non-small cell lung cancer cells. Human breast MCF7, SKBR3, T47D, MDA-MB-231 and human non-small cell lung cancer NCI-H1395, NCI-H1650, NCI-H1975 and A549 cells were lysed and probed with the indicated antibodies by immunoblotting. Tubulin was used as control of equal protein loading. The image is representative of 1 out of 3 experiments.

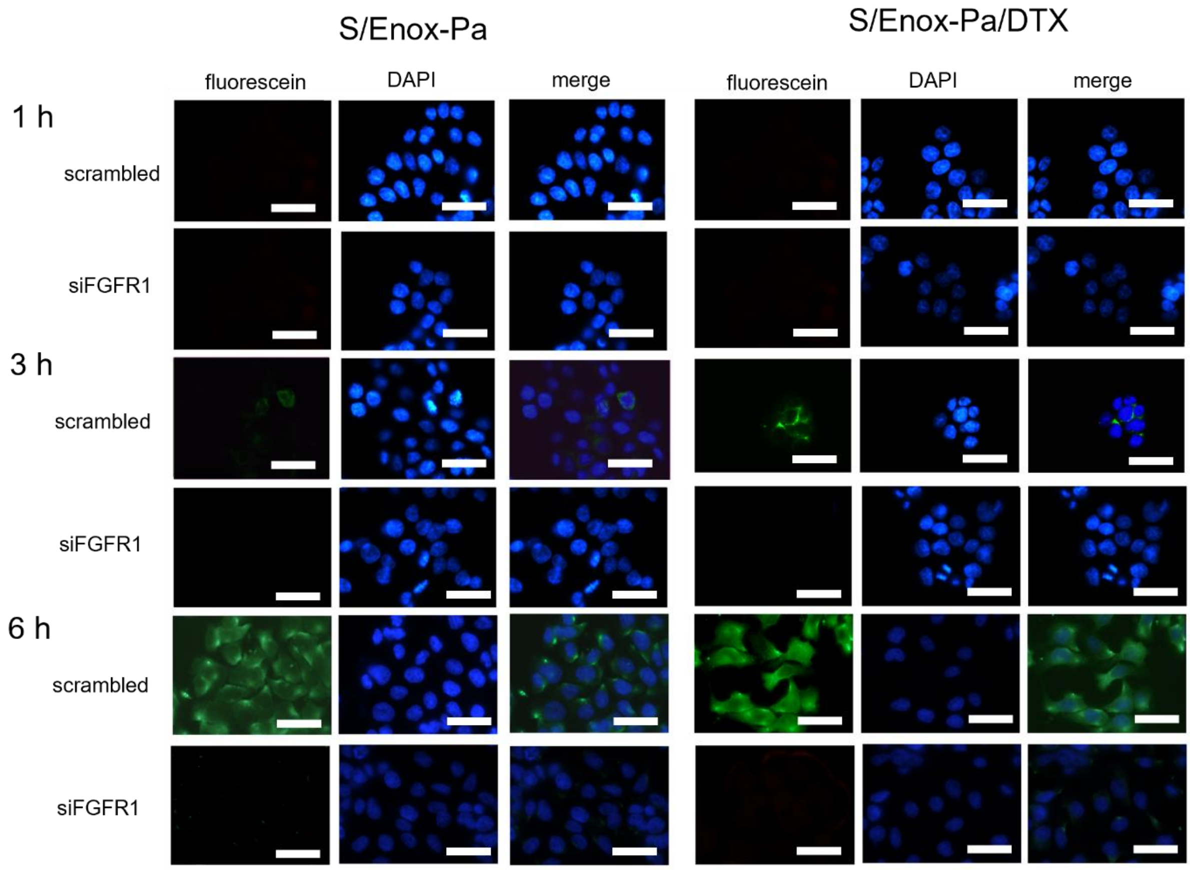

Figure S2. Time-dependent intracellular accumulation of Enoxaparin-conjugated SEDDS. A549 cells were treated 48 h with a non-targeting siRNA (scrambled) or with a pool of 3 siRNAs targeting FGFR1 (siFGFR1), then incubated 1, 3 or 6 h with 0.25% v/v blank SEDDS (S), enoxaparin-conjugated SEDDS (S/Enox-Pa), or enoxaparin-conjugated SEDDS containing docetaxel (80 μM final concentration; S/Enox-Pa/DTX). Representative photographs of scrambled and siFGFR1 A549 cells at each time point were shown. The photos are representative of 1 out of 3 experiments. For each experimental condition a minimum of 5 field were examined. The photos are representative of 1 out of 3 experiments. Ocular: 10X; objective: 60X. Bars: 50 μm.

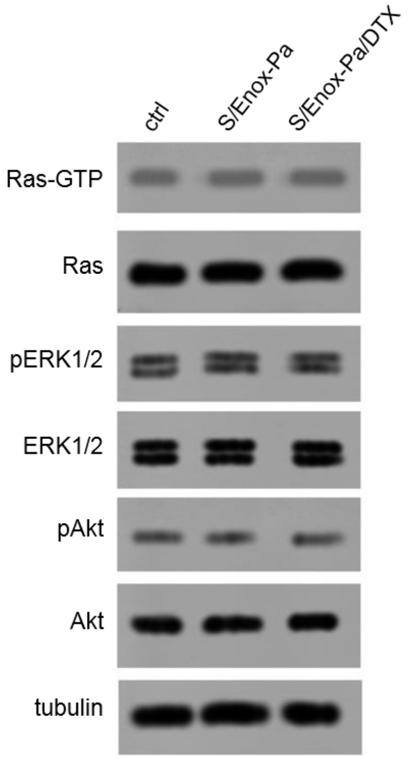

Figure S3. Expression of downstream effectors of FGFR1 in A549 cells treated with Enox-coated SEDDS. A459 cells were incubated for 24 h with fresh medium (ctrl), 0.25% v/v Enox coated SEDDS (S/Enox-Pa) or Enox coated SEDDS containing docetaxel (80 µM final concentration; S/Enox-Pa/DTX), then lysed and probed with the indicated antibodies by immunoblotting. Tubulin was used as control of equal protein loading. The image is representative of 1 out of 3 experiments.

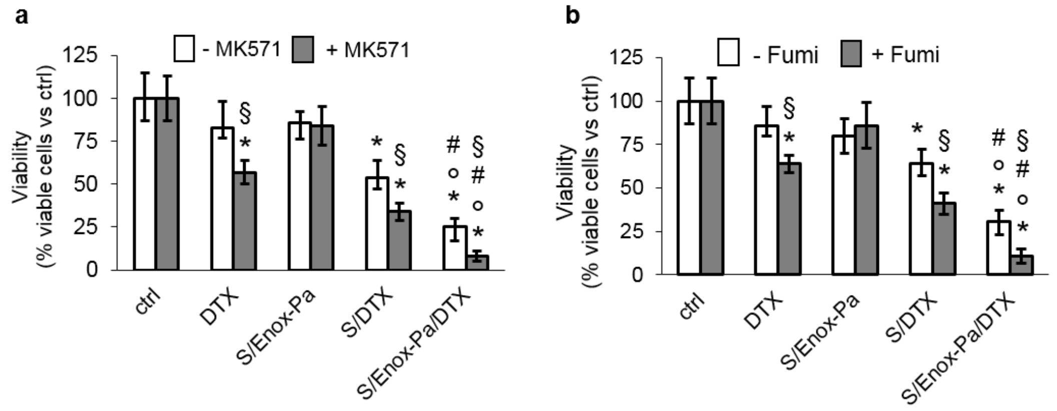

Figure S4. Effects of MRP1 and BCRP pharmacological inhibition on Enox-coated SEDDS containing DTX. A459 cells were incubated for 72 h with fresh medium (ctrl), 80 µM free docetaxel (DTX), 0.25% v/v Enox coated SEDDS (S/Enox-Pa), SEDDS containing docetaxel (80 µM final concentration; S/DTX), Enox coated SEDDS containing docetaxel (80 µM final concentration; S/Enox-Pa/DTX), in the presence of the MRP1 inhibitor MK571 (25 µM; panel a) or BCRP inhibitor fumitremorgin (5 µM, Fumi; panel b). Cell viability was measured by a chemiluminescence-based assay in quadruplicates. Data are presented as means + SD (n=3). *p<0.001: vs ctrl; °p<0.001: S/Enox-Pa/DTX vs DTX; #p<0.01: S/Enox-Pa/DTX vs S/DTX; §p<0.01: MK571/Fumitremorgin-treated (+) vs untreated (-) cells.

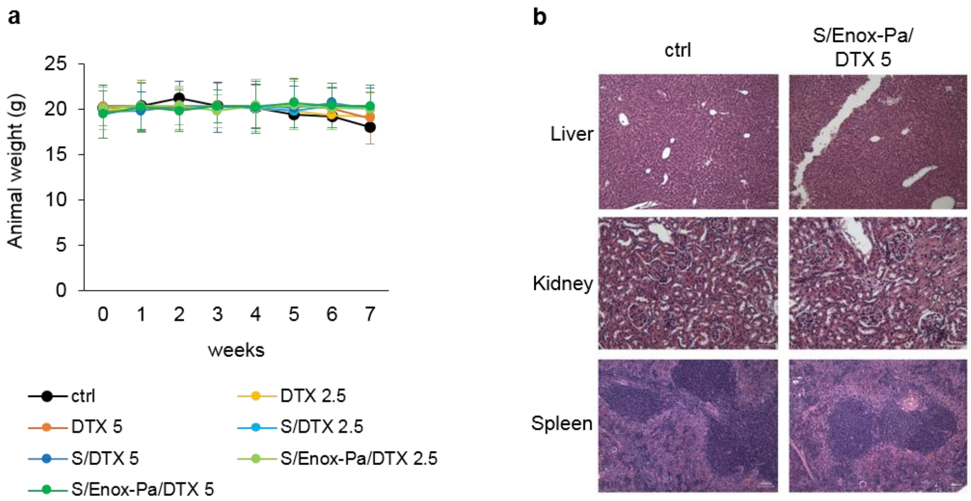

Figure S5. Weight monitoring and post-mortem tissue examination. 1 x 10^6 A549 cells were inoculated subcutaneously in the right flank of 6-week-old Balb/C female nude mice. When tumors reached the volume of 100 mm^3, mice (n= 8/group) were randomized in the following groups and treated once a week for 6 weeks as reported: 1) Vehicle group (ctrl), with 100 µL saline solution intravenously (i.v.); 2) docetaxel 2.5 mg kg^{-1} (DTX 2.5), in 100 µL Intralipid i.v.; 3) docetaxel 5 mg kg^{-1} (DTX 5), in 100 µL Intralipid i.v.; 4) SEDDS containing docetaxel at 2.5 mg kg^{-1} final concentration (S/DTX 2.5) i.v.; 5) SEDDS containing docetaxel at 5 mg kg^{-1} final concentration (S/DTX 5) i.v.; 6) Enox coated SEDDS containing docetaxel at 2.5 mg kg^{-1} final concentration (S/Enox-Pa/DTX 2.5) i.v.; 7) Enox coated SEDDS containing docetaxel at 5 mg kg^{-1} final concentration (S/Enox-Pa/DTX 5), i.v. Animals were euthanized at week 7. (a) Animals weight was monitored weekly. (b) Representative hematoxylin-eosin staining of liver, kidney and spleen examined post-mortem in ctrl and S/Enox-Pa/DTX 5 group. For each experimental condition a minimum of 5 field were examined. Ocular: 10X; objective: 10X (liver, spleen), 20X (kidney). Scale bar: 50 µm.

Table S1. Hematochemical parameters of the animals.

3.5 weeks	ctrl	DTX 2.5	DTX 5	S/DTX 2.5	S/DTX 5	S/Enox-Pa/ DTX 2.5	S/Enox-Pa/DTX 5
RBC (x 10^6 μL^{-1})	14.15 ± 1.45	12.09 ± 3.05	11.24 ± 1.02	12.34 ± 2.87	11.34 ± 0.89	13.44 ± 3.09	13.88 ± 1.13
Hb (g dL^{-1})	13.01 ± 1.49	12.22 ± 2.07	11.08 ± 0.98	12.74 ± 1.93	10.98 ± 1.05	12.54 ± 2.08	12.23 ± 1.29
WBC (x 10^3 μL^{-1})	17.82 ± 2.36	15.78 ± 3.45	14.89 ± 1.78	16.02 ± 2.67	15.32 ± 1.62	16.78 ± 2.17	16.09 ± 0.94
PLT (x 10^3 μL^{-1})	983 ± 304	789 ± 289	675 ± 193	834 ± 309	705 ± 234	978 ± 209	859± 372
LDH (U L^{-1})	7349 ± 609	7672 ± 709	7029 ± 678	6892 ± 508	6783 ± 537	6213 ± 302	6054 ± 432
AST (U L^{-1})	204 ± 45	189 ± 37	213 ± 42	176 ± 38	198 ± 38	152 ± 39	212 ± 31
ALT (U L^{-1})	39 ± 10	44 ± 11	36 ± 9	38± 9	40 ± 11	43± 11	37 ± 8
AP (U L^{-1})	99 ± 25	107 ± 17	98 ± 23	87 ± 13	101 ± 34	93 ± 11	98 ± 26
Creatinine (mg L^{-1})	0.069 ± 0.014	0.074 ± 0.009	0.071 ± 0.01	0.075 ± 0.011	0.069 ± 0.009	0.068 ± 0.010	0.068 ± 0.012
CPK (U L^{-1})	267 ± 61	209 ± 74	238 ± 47	281 ± 39	273 ± 72	278 ± 13	234 ± 45

7 weeks	ctrl	DTX 2.5	DTX 5	S/DTX 2.5	S/DTX 5	S/Enox-Pa/ DTX 2.5	S/Enox-Pa/DTX 5
RBC (x 10^6 μL^{-1})	14.01 + 1.13	11.74 + 1.89	10.87 + 1.56 *	11.65 + 2.17	10.44 + 1.67 *	13.82 + 1.82	12.04 + 1.55
Hb (g dL^{-1})	12.82 + 0.92	11.29 + 1.29	10.45 + 0.76 *	11.08 + 2.39	10.02 + 0.88 *	12.2 + 1.73	11.29 + 1.48
WBC (x 10^3 μL^{-1})	17.14 + 2.11	13.18 + 2.87	13.12 + 0.98 *	13.82 + 1.78	13.88 + 1.43 *	16.27 + 1.98	15.45 + 1.82
PLT (x 10^3 μL^{-1})	982 + 201	678 + 293	578 + 209 *	713 + 304	589 + 233 *	883 + 283	785 + 238
LDH (U L^{-1})	10892 + 654	9623 + 453	9783 + 563	7987 + 562	6754 + 398	6521 + 394	6412 + 402
AST (U L^{-1})	189 + 29	218 + 44	198+ 45	193 + 49	210+ 76	188 + 34	198 + 87
ALT (U L^{-1})	41 + 11	41 + 15	38 +12	42 + 9	41 + 11	37 + 11	38 + 10
AP (U L^{-1})	101 + 34	96 + 23	103 + 24	102 + 12	89 + 17	89 +9	112 + 18
Creatinine (mg L^{-1})	0.075 + 0.011	0.072 + 0.012	0.07 + 0.008	0.065 + 0.007	0.074+ 0.005	0.077 + 0.012	0.069+ 0.011
CPK (U L^{-1})	244 + 53	214 + 28	245 + 86	238 + 78	238 + 76	247 + 55	273 + 37

Animals were subjected to the experimental protocol of Figure 4 and S4. At 3.5 and 7 week (i.e. at the mid-point and at the end of the study), 200 μL blood were collected to measure the following parameters: red blood cells (RBC), white blood cells (WBC), haemoglobin (Hb), platelets (PLT), lactate dehydrogenase (LDH), aspartate aminotransferase (AST), alanine aminotransferase (ALT), alkaline

phosphatase (AP), creatine phosphokinase (CPK). Data are presented as means + SD (n=8 animals/group). *p<0.05: vs ctrl.
